# Manipulation of the Upper Respiratory Microbiota to Reduce Incidence and Severity of Upper Respiratory Viral Infections: A Literature Review

**DOI:** 10.3389/fmicb.2021.713703

**Published:** 2021-08-27

**Authors:** Henry Nesbitt, Catherine Burke, Mehra Haghi

**Affiliations:** ^1^Discipline of Pharmacy, Graduate School Health, University of Technology Sydney, Sydney, NSW, Australia; ^2^School of Life Sciences, University of Technology Sydney, Sydney, NSW, Australia

**Keywords:** respiratory, microorganism, virus, bacteria, microbiota, commensal, pathobiont, dysbiosis

## Abstract

There is a high incidence of upper respiratory viral infections in the human population, with infection severity being unique to each individual. Upper respiratory viruses have been associated previously with secondary bacterial infection, however, several cross-sectional studies analyzed in the literature indicate that an inverse relationship can also occur. Pathobiont abundance and/or bacterial dysbiosis can impair epithelial integrity and predispose an individual to viral infection. In this review we describe common commensal microorganisms that have the capacity to reduce the abundance of pathobionts and maintain bacterial symbiosis in the upper respiratory tract and discuss the potential and limitations of localized probiotic formulations of commensal bacteria to reduce the incidence and severity of viral infections.

## Introduction

The upper respiratory tract (URT) is the epicenter of the respiratory microbiota. As a “portal of entry” into the respiratory system, the URT’s proximity to the external environment allows for adherence and colonization of a diverse and abundant microbiota. A healthy upper respiratory microbiota works in synergy with its host, mainly colonizing the anterior nares and nasopharynx to provide an innate barrier that defends against pathogens and modulates immune responses that occur from exposure to external triggers (Man et al., 2017; Hakansson et al., 2018). These external triggers include smoke, dust, allergens, chemical irritants, changes in temperature, and microorganisms (Burbank et al., 2017).

A variety of bacteria are found in the URT including commensals that are thought to promote a healthy epithelium, but also pathobionts that can be benign or pathogenic under certain circumstances. The most prevalent commensals include *Corynebacterium* spp., *Dolosigranulum pigrum*, *Streptococcus mitis/oralis, Staphylococcus epidermis*, and *Haemophilus haemolyticus*, while pathobionts include *Streptococcus pneumoniae*, *Haemophilus influenzae*, *Staphylococcus aureus*, and *Moraxella catarrhalis* (Escapa et al., 2018). Viral infections can enable pathobionts to initiate secondary infections by damaging epithelial cells and inhibiting mucociliary clearance (Ahern and Cervin, 2019). However, several cross-sectional studies analyzed in the literature indicate that an inverse relationship may also occur, whereby pathobiont abundance and/or bacterial dysbiosis could cause impairment of epithelial integrity and predispose an individual to viral infection (Pitkaranta et al., 2006; Moore et al., 2010). Therefore, this review looks at the current research surrounding the URT microbiota, its influence on viral infection and the potential role of commensal bacteria in the prevention and management of viral upper respiratory infections.

## The Airway Epithelial Barrier and Innate Responses to Microorganisms

Apart from its function in facilitating gas exchange, the airway epithelium also acts as a physical and chemical barrier against infection from microorganisms. This is achieved through the combined action of the mucociliary escalator and the maintenance of a tight physical barrier. Mucus is produced by goblet cells and can trap and neutralize microorganisms via mucins, antimicrobial proteins and immunoglobulins secreted from epithelial cells. Cilia on the apical surface of ciliated cells beat synchronously to move mucus and the microorganisms trapped within, away from the airways to expel them (Bergelson, 2009; Voynow and Rubin, 2009). The physical barrier is formed via proteins that promote tight cell-cell adhesion of epithelial cells including tight junctions (TJs), adherent junctions (AJs), gap junctions (GJs), and desmosomes (Rezaee and Georas, 2014). They form an impenetrable barrier preventing viral and bacterial entry through the epithelial layer, systemic spread through the circulation and access to viral receptors on the basolateral epithelial surface (Bergelson, 2009; Voynow and Rubin, 2009; Sharma et al., 2020).

Epithelial and resident sensor cells including macrophages and dendritic cells can sense and respond to the presence of microorganisms via pattern recognition receptors (PRRs). PRRs expressed by the respiratory epithelium include Toll-Like Receptors (TLRs), epidermal growth factor (EGF) and C-type lectins. PRRs recognize conserved microbial molecules such as components of bacterial and fungal cell walls, flagellin, viral RNA, as well as host cell components that indicate cell damage (Parker and Prince, 2011). Sensing of bacterial, fungal and viral components initiates the release of signaling molecules (cytokines and chemokines) that drive the innate immune response (Invernizzi et al., 2020). For example, stimulation of PRRs can modulate intercellular junctions (including TJs, GJs, AJs, and desmosomes) through the upregulation of proinflammatory cytokines or epidermal growth factor (EGF) which can result in the weakening or strengthening of the respiratory epithelial barrier, respectively (Lebeer et al., 2010; Martens et al., 2018).

Cytokines can act locally on epithelial cells to upregulate the expression of genes that contribute to pathogen clearance, like mucus production, antimicrobial peptides and interferons (INFs) (Parker and Prince, 2011). Cytokines and chemokines also activate and recruit immune cells that perform a range of control mechanisms including phagocytosis and inflammation (Invernizzi et al., 2020). Interferons are particularly important for the control of viruses, as they signal the presence of a viral infection to surrounding cells and upregulate genes that restrict viral replication (Fensterl and Sen, 2009). These innate immune responses help protect against pathogen infection, however, there are clearly different responses to commensal versus pathogenic microorganisms that enable the respiratory microbiota to colonize the epithelium without chronically stimulating an inflammatory immune response.

While the microbiota is in part controlled by exclusion from the epithelium via the mechanisms described above, there is also evidence that commensals can directly stimulate immune tolerance and inhibit inflammatory signaling. For example, in the gut the commensal *Bacteroidetes thetaiotaomicron* can inhibit NF-κB expression in intestinal epithelial cells (Kelly et al., 2004) and the production of short chain fatty acids (SCFA) by *Clostridium* spp. stimulates the expansion of anti-inflammatory T-regulatory cells (Tregs) (Atarashi et al., 2011). Tregs are important modulators of immune tolerance, and their expansion and development is also stimulated directly via antigen recognition of specific commensal bacteria (Russler-Germain et al., 2017), suggesting the involvement of both innate and adaptive immune mechanisms in tolerance to the microbiota. These examples come from the gut, which has been more extensively characterized for host microbiota interactions, but it is likely that similar mechanisms exist in the URT. While research on immune stimulation by respiratory commensals is scarce, studies with traditional probiotic strains of bacteria like *Lactobacillus* spp. have shown they can stimulate expansion of Tregs via contact with dendritic cells, resulting in increased expression of anti-inflammatory cytokines such as interleukin (IL)-10, and inhibition of proinflammatory cytokines including IL-2, IL-4, IL-5 and tumor necrosis factor alpha (TNF-α) (Martens et al., 2018). Commensals native to the URT are likely to similarly stimulate immunotolerance, suggesting it is possible to tune the host inflammatory state via manipulation of the resident microbiota.

## The URT Microbiota

The URT microbiota is dominated by bacteria from Actinobacteria and Firmicutes phyla, with smaller proportions of species from the Proteobacteria and Bacteroidetes. The URT is colonized by diverse communities of microorganisms, with changes in community structure associated with different anatomical locations and epithelial types (Yan et al., 2013; Proctor and Relman, 2017). The anterior nares are closest to the external environment and are lined with keratinized squamous epithelium, and sebaceous glands that secrete the host derived lipid and sebum (Man et al., 2017), while the sino-nasal and nasopharyngeal mucosa has a pseudostratified columnar and ciliated epithelium that produces mucus (Beule, 2010).

*Cutibacterium* (previously *Propionibacterium*) and *Corynebacterium* spp. are lipophilic skin colonizers which along with *Staphylococcus* spp. commonly dominate in the anterior nares, while the nasal mucosa supports a greater diversity of bacteria including *Moraxella*, *Dolosigranulum* and *Streptococcus* spp. (Yan et al., 2013; Man et al., 2017). The nasopharynx contains patches of scattered respiratory epithelial cells but is mainly lined with stratified squamous epithelium (Man et al., 2017), like the nasal mucosa there are more abundant and diverse bacterial communities in the nasopharynx in comparison to the anterior nares (Yan et al., 2013). *Dolosigranulum*, *Haemophilus* and *Streptococcus* spp. are frequent colonizers of the nasopharynx which also commonly contains *Moraxella*, *Corynebacterium*, and *Staphylococcus* spp. (Man et al., 2017).

### Development of the URT Microbiota

In the development of the URT microbiota, mode of delivery and type of infant feeding play a key role in the development of bacterial diversity and abundance (Esposito and Principi, 2018). Dominant organisms from the anterior nares (*Staphylococcus*, *Corynebacteria*, and *Cutibacterium* are thought to be acquired via skin to skin contact (Esposito and Principi, 2018). These are also dominant taxa from the skin microbiota which is in close proximity to the anterior nares, suggesting that the microbiota of the skin influences the URT microbiota. Breast-fed infants were shown to have an increased abundance of *Corynebacterium* spp. in their URT in comparison to formula fed infants who showed an *S. aureus* dominated bacterial profile (Biesbroek et al., 2014a). Maternal breast milk has its own microbiota, in which *Corynebacterium* spp. are frequently detected, indicating that along with skin contact, breast feeding is another source of colonization with this taxa within the first few months of life (Zimmermann and Curtis, 2020).

*D. pigrum* is also abundant, but in lower quantities in comparison to the dominant three. *D. pigrum* is obtained in early development of the human upper respiratory microbiota, likely from vaginal microbiota acquired from a vaginal delivery (Esposito and Principi, 2018). An increased abundance of *D. pigrum* in infants was found to be associated with vaginal delivery as opposed to infants born by cesarean section and to be more abundant in the nasopharynx than in the anterior nares (Bosch et al., 2016; De Boeck et al., 2017). *D. pigrum* can produce lactic acid giving it the potential to lower the pH of the local environment which may select for *Corynebacterium* spp. growth, potentially explaining their co-occurrence within the upper respiratory tract (De Steenhuijsen Piters et al., 2015). *Haemophilus* spp. and *Moraxella* spp. also colonize the URT in early development, however, the way they are acquired is not completely understood. In healthy development they have been shown to be particularly abundant in pre-schoolers in comparison to younger infants and older children (Bae et al., 2012). This may give some indication as to the time they are inoculated into the URT microbiota. The development of the URT microbiota in infancy is an important predictor of the frequency of respiratory infections in children (Teo et al., 2018; Dubourg et al., 2019) and may continue to play a role in respiratory health and disease later in life.

## The Influence of Pathobionts on URT Viral Infection

URT infections (URTIs) include non-allergic rhinitis (the common cold), rhinosinusitis, pharyngitis, tonsillitis and otitis media. URTIs are a very common problem, especially among infants, children and elderly, and are one of the most frequent presentations in general practice (Cooke et al., 2013). URTIs can be caused by viruses or bacteria, however, viral infections are the more dominant cause. Upper respiratory viruses that cause both rhinitis and/or sinusitis include human rhinovirus, respiratory syncytial virus (RSV), influenza and parainfluenza viruses, coronaviruses, adenoviruses and enteroviruses (Thomas and Bomar, 2021). While the ability of respiratory viruses to enable subsequent bacterial co-infections has been well established (Bakaletz, 2017), current evidence suggests that the inverse may also occur. The expansion of different pathobionts in the URT microbiota may increase the incidence and severity of URT viral infections (Bosch et al., 2013).

Dominance of a pathobiont in the URT microbiota can be considered as dysbiosis, which can be defined as either a loss of commensal microbes, the proliferation of pathobionts or a loss of total microbial diversity (Martens et al., 2018). Dysbiosis has been associated with impending, recurrent, and chronic disease (Man et al., 2017; Wilkins et al., 2019). URT dysbiosis could be caused by changes to the URT environment such as inflammation or the use of antibiotics. Oral antibiotics have a significant impact on the gut microbiota (Schwartz et al., 2020), however, the effect on the URT microbiota is less clear. The concentration of antibiotic in the URT mucosa is likely to be lower than in the gut (Siu et al., 2019), and the reported effects of antibiotics on the URT are varied including increases (Merkley et al., 2015) or decreases (Liu et al., 2013) in microbial diversity, or no significant effects at all (Siu et al., 2021). Interpretation of these studies is further complicated by differences in treatment and disease status of the subjects. The possibility that antibiotics or other medical treatments like steroids could cause dysbiosis and proliferation of pathobionts is an area for further study.

Pathobionts are defined as bacteria that are commonly found in healthy asymptomatic individuals, but that can also be pathogenic under certain conditions. *S. pneumoniae*, *H. influenzae*, *S. aureus*, and *M. catarrhalis* have been identified as bacterial pathobionts and an increased abundance of one or more of these are often features of dysbiosis in the URT (Bosch et al., 2013). An increased abundance of pathobionts often leads to a decrease in microbiota diversity, which is hypothesized to contribute to a susceptible innate epithelial barrier and increased inflammation in response to environmental triggers including respiratory viruses (Esposito and Principi, 2018).

**FIGURE 1:**
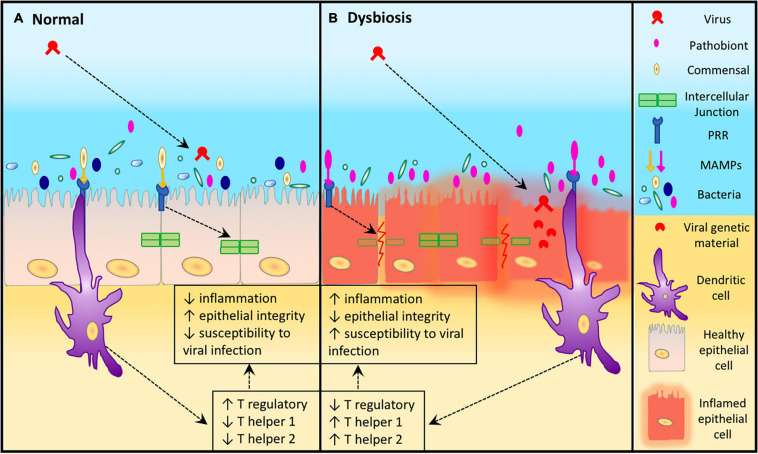
Epithelial susceptibility to viruses during dysbiosis. **(A)** In a healthy URT where there is a diverse microbiota, higher numbers of commensal Microorganism-Associated Molecular Patterns (MAMPs) are able to attach to the Pattern Recognition Receptors (PRRs) of epithelial and dendritic cells resulting in maintenance of epithelial integrity and reduced susceptibility to viral infection. **(B)** Dysbiosis in the URT leads to an increase abundance of a pathobiont which increases the attachment of pathobiont MAMPs to the PRRs of epithelial and dendritic cells resulting in increased inflammation, reduced epithelial integrity. This leads to host cell damage which increases the susceptibility of host URT to viral infection.

Several mechanisms could explain the association of bacterial dysbiosis and viral infections. URT pathobionts can secrete products that impair ciliary action, reducing the capacity for mucociliary clearance (Janson et al., 1999; Shen et al., 2012). Secreted bacterial products (e.g., elastase) can also directly impact TJ proteins, reducing epithelial barrier function (Malik et al., 2015; Li et al., 2019). Alternatively, sensing pathobionts via TLRs can also downregulate TJ protein expression (Clarke et al., 2011). Disruption of barrier function may lead to increased accessibility of viral particles to the basolateral surface as an alternative entry point. Additionally, some pathobionts are known to upregulate the expression of viral receptor proteins in epithelial cells (Frick et al., 2000). These mechanisms are plausible ways by which the presence or increased abundance of pathobionts may help facilitate viral infections, above and beyond the ability of a virus to overcome the host’s innate immune defenses. An overview of these mechanisms is illustrated in Figure 1.

In the following sections we will summarize some of the known relationships where URT pathobionts enable or exacerbate infections by respiratory viruses.

### Respiratory Syncytial Virus (RSV)

RSV is a frequent cause of bronchiolitis in young children and older adults (Stensballe et al., 2006; Tin Tin Htar et al., 2020). RSV infects ciliated epithelial cells via binding of its G-protein with the receptor CX3CR1 (Tin Tin Htar et al., 2020). During *in vitro* co-infection *S. pneumoniae* upregulates bacterial proteins such as superoxide dismutase, thioredoxin and histone-like DNA binding protein (hlpA) which protect *S. pneumoniae* against oxidative stress (Shadia et al., 2019). The protein hlpA, forms soluble antigen complexes with lipoteichoic acid that bind to epithelial cells and induce a proinflammatory cascade in the upper respiratory tract (Stinson et al., 1998). *S. pneumoniae* and RSV dominant profiles have been shown to be associated with greater levels of lipoteichoic acid (Chonmaitree et al., 2017). This inflammatory cascade results in an increased production of IL-6 and IL-8. These cytokines contribute to macrophage signaling and neutrophil recruitment which is associated with more severe symptoms of upper respiratory infections caused by RSV (Gulraiz et al., 2015). Inversely, gene expression in *S. pneumoniae* is affected by the presence of RSV, including an increase in the expression of virulent genes such as the pneumococcal toxin, pneumolysin, which is associated with virulent strains of *S. pneumoniae* (Smith et al., 2014). *S. pneumoniae* and RSV coinfection contributes to delayed recovery and indicates a synergism between the two microorganisms with negative consequences for the host (Brealey et al., 2018).

### Human Rhinovirus (HRV)

HRV is the most frequent cause of the common cold, and a common exacerbator of chronic respiratory diseases such as COPD and asthma (Jacobs et al., 2013; Blaas and Fuchs, 2016). *H. influenzae* has been found to increase the expression of the HRV receptor, intercellular adhesion molecule 1 (ICAM-1) in epithelial cells (Gulraiz et al., 2015). Upregulated ICAM-1 in respiratory epithelial cells increases the sensitivity of basolateral cells to HRV infection (Blaas and Fuchs, 2016). As a result, *H. influenzae* promotes higher viral loads of HRV therefore enhancing the inflammatory response (Gulraiz et al., 2015). Individuals with respiratory viruses and a high abundance of *H. influenzae* were found to suffer from more severe symptoms and increased radiological findings than with viral infection alone (Autio et al., 2015). In particular HRV was shown to be associated with a microbiota with a high relative abundance of *H. influenzae* further reinforcing their relationship (Jacoby et al., 2007; Van Den Bergh et al., 2012).

### Influenzae Virus

*S. aureus* has long been observed in co-infections with influenza virus. Influenza is known to promote *S. aureus* infection by modulation of the immune system (e.g., by depleting phagocytic cells; Ghoneim et al., 2013) and increasing *S. aureus* adherence and internalization into host cells (Passariello et al., 2011). This relationship goes both ways with *S. aureus* also enhancing influenza replication and infection severity, for example by the secretion of staphylokinase which enhances viral binding to host cells (Scheiblauer et al., 1992). Strains of *S. aureus* can produce enterotoxins A and B, that can disable cilia and therefore decrease mucociliary clearance (Min et al., 2006). This can lead to a host immune response, initiating the production of M1 alveolar macrophages (Hakansson et al., 2018). This in turn increases proinflammatory cytokines, TNF-α and IL-1β, and apoptosis of respiratory cells resulting in increased susceptibility to influenzae viral invasion into respiratory tissue. Inflammatory responses to influenzae infection and resulting URT symptoms such as rhinitis are likely heightened as a result of the increased bacterial load of *S. aureus* in the URT, which may occur in respiratory dysbiosis (Hakansson et al., 2018).

### Adenovirus/Coronavirus

An increased abundance of *M. catarrhalis* has been positively associated with occurrence of Adenovirus or Coronavirus (Van Den Bergh et al., 2012). These three microorganisms are commonly associated as predominant causes of otitis media (Moore et al., 2010). There currently isn’t much evidence supporting a symbiotic relationship in the literature. *M. catarrhalis* is known to utilize immunoglobulin D and hemagglutinin for the stimulation of high-density IgD-bearing B lymphocytes causing a proinflammatory response (De Vries et al., 2009). Also *M. catarrhalis’* MAMPs can increase the expression of TLR-2 and subsequent transcription of proinflammatory genes (De Vries et al., 2009). TLR-2 upregulation also leads to a downregulation of IL-8 and a reduction in degranulation and chemotaxis of neutrophils which may increase the ability of Adenoviruses and Coronaviruses to adhere to respiratory epithelial cells and allow for a more severe URT viral infection due to a decreased neutrophilic response from the host.

There have been several studies examining correlations between the URT microbiota and SARS-CoV2 infection since the beginning of the COVID-19 global pandemic. These studies indicate that the microbiota is shifted with an enrichment of pathobionts and opportunistic pathogens in COVID-19 patients compared to non-infected individuals (Engen et al., 2021; Merenstein et al., 2021; Rhoades et al., 2021). We note that at the time of writing, some of these studies have not yet been peer reviewed. The microbiota was only sampled after infection with SARS-CoV2 was identified, so we don’t yet know whether pathobiont presence or expansion could increase the risk of SARS-CoV2 infection, or if the infection itself might drive a microbiota shift. However one study found that several *Streptococcus* spp. increase the expression of the ACE2 receptor protein in mammalian cells, indicating a possible mechanism by which pathobionts could influence the risk of infection (Xiong et al., 2021).

All of the examples given above describe ways in which pathobionts may increase the risk or severity of a viral infection. The ability to prevent or limit the colonization of the URT by these pathobionts could therefore represent a viable strategy to reduce the risk of respiratory viral infection.

## Commensal Bacteria’s Role in Maintaining a Healthy URT Microbiota

*D. pigrum*, *Corynebacterium* spp., *S. epidermis*, *S. mitis/oralis*, and *H. haemolyticus* are commonly found in the URT microbiota and many observational studies have found associations with these bacteria and decreased risk or incidence of URT infections (see references provided below). *In vitro* studies have shown the ability of these URT colonizers to inhibit the growth of pathobionts (see references provided below), and a few human studies show the potential for this to occur in the respiratory tract (Uehara et al., 2000; Iwase et al., 2010; Kiryukhina et al., 2013). The ability to inhibit pathobionts could reduce the risk of viral respiratory infections given the evidence described above. Commensal respiratory bacteria could also influence the risk of viral infection through modulation of the host immune system or even through interaction with the virus itself (Dragelj et al., 2021). An overview of the evidence regarding interactions between commensal bacteria and pathobionts as well as the host immune system is given below and has been summarized in Table 1.

**TABLE 1:** Association between commensal bacteria and bacterial pathobionts in the URT.

**Commensal**	**Pathobiont**	**Association**	**Mechanism**	**Study**	**References**
*D. pigrum*	*S. aureus*	−	Lanthipeptide and/or bacteriocins	*In vitro* and *in vivo* human	Liu et al., 2015; Brugger et al., 2020
*D. pigrum* and *Corynebacterium* spp.	*S. pneumoniae*	−	Free fatty acid accumulation and host immune modulation	*In vitro* and *in vivo* human	Schenck et al., 2016; Lappan and Peacock, 2019
*C. pseudodiptheriticum*	*M. catarrhalis*	−	Host immune modulation	*In vitro*	Lappan and Peacock, 2019
	*S. aureus*	−	Competition for nutrients	*In vitro* and *in vivo* infant mice	Kiryukhina et al., 2013; Yan et al., 2013
*C. accolens*	*S. pneumoniae*	−	Triolein	*In vitro*	Bomar et al., 2016
	*S. aureus*	+	Commensalism	*In vitro*	Yan et al., 2013
*S. salivarius*	*S. pneumoniae*	−	Blocks pneumococcal binding sites	*In vitro*	Manning et al., 2016
*S. salivarius and S. oralis*	*S. aureus, S. pneumoniae and M. catarrhalis*	−	Biofilm degradation	*In vitro*	Bidossi et al., 2018
	*M. catarrhalis*	−	Competence Stimulating Peptides (CSP)	*In vitro* and *in vivo* human	De Grandi et al., 2019
*S. epidermis*	*S. aureus*	−	Extracellular serine proteases	*In vitro* and *in vivo* human	Iwase et al., 2010
*H. haemolyticus*	*H. influenzae*	−	Bacteriocin like substance	*In vitro*	Latham et al., 2017
		−	Haemophilin	*In vitro*	Atto et al., 2020

### *Dolosigranulum pigrum* and *Corynebacterium* spp.

Species within the *Dolosigranulum* and *Corynebacterium* genera have been associated with decreased rates of pathobionts and URT infections in a range of studies. Potential mechanisms include direct inhibition via production of antimicrobial compounds and competition for nutrients, and indirect inhibition via stimulation of the host immune system (De Steenhuijsen Piters and Bogaert, 2016; Lappan and Peacock, 2019).

In one longitudinal study, children resistant to acute otitis had a significantly higher abundance of *D. pigrum* and *Corynebacterium* spp. in their nasopharynx in comparison to children who suffered from acute otitis media (Lappan et al., 2018). Metagenomics of the resistant children further revealed *C. pseudodiphtheriticum* and *D. pigrum* to be dominant species in the nasopharynx of the resistant children, with *C. propinquum* and *C. accolens* present to a lesser extent (Lappan, 2019). In fact, correlations have been found in many observational studies where decreased relative abundance of *Corynebacterium* spp. and *Dolosigranulum* spp. was associated with increased risk of respiratory infections (Laufer et al., 2011; Biesbroek et al., 2014a,b), wheezing (Biesbroek et al., 2014a), symptomatic viral infections (Kloepfer et al., 2017), chronic rhinosinusitis (Cleland et al., 2016; Copeland et al., 2018), cystic fibrosis (Prevaes et al., 2016), and was inversely correlated with *S. pneumoniae* colonization (Bomar et al., 2016). While there is some disparity in the literature, the majority of studies have found a negative correlation between *Corynebacterium* spp. and *S. aureus* relative abundance or carriage (Uehara et al., 2000; Lina et al., 2003; Yan et al., 2013) suggesting at least some species in this genus may be able to inhibit *S. aureus* colonization in the nose. There is thus a wealth of observational evidence to suggest that *Corynebacterium* and/or *Dolosigranulum* spp. in the respiratory microbiota may be beneficial.

*In vitro* evidence exists to support the ability of *Corynebacterium* spp. to inhibit the growth and colonization of pathobionts in the respiratory tract. *C. accolens* can inhibit *S. pneumoniae* via liberation of free fatty acids from triacylglycerols found on the skin (Bomar et al., 2016), and clinical isolates of *C. accolens* can inhibit the growth of methicillin resistant *S. aureus* (Menberu et al., 2021). *C. pseudodiphtheriticum* is inhibitory against *M. catarrhalis* and *S. aureus* (Hardy et al., 2019; Lappan, 2019). Likewise, *D. pigrum* has been observed to inhibit both *S. aureus* and *S. pneumoniae in vitro*, in the latter case requiring spent media from *Corynebacterium* spp. for inhibition to occur (Brugger et al., 2020). Other mechanisms like downregulation of *S. aureus* virulence genes when co-cultured with *Corynebacterium striatum* have also been observed (Ramsey et al., 2016).

Apart from effects on the growth of pathobionts, *Corynebacterium* spp. may also mediate beneficial effects by modulation the immune system. Indirect evidence from humans suggests that *Corynebacterium* spp. may stimulate IFN-γ (De Steenhuijsen Piters and Bogaert, 2016) and this has also been observed in a mouse model where nasal inoculation with *C. pseudodipthereticum* increased resistance to RSV infection (Kanmani et al., 2017). IFN-γ stimulates antiviral functions in T-cells and natural killer cells. Induction of IFN-γ could explain the decreased risk of upper respiratory viral infections when *Corynebacterium* spp. are in higher abundance in the URT microbiota (De Steenhuijsen Piters and Bogaert, 2016).

Similarly *D. pigrum* and *C. pseudodiptheriticum* can modulate the immune response in mice. Nasal administration of both species was shown to increase levels of the antiviral cytokines IFN-β and IFN-γ, and the anti-inflammatory cytokine IL-10, however, only particular strains of these species had an effect. Further experiments with *D. pigrum* showed this effect was associated with reduced lung damage markers and increased resistance to RSV infection (Ortiz Moyano et al., 2020). The same strain of *D. pigrum* was also found to increase expression of IFN-β as well as IL-6 in a bronchial epithelial cell line (Calu-3) which was associated with reduced viral titres of SARS-CoV-2 and a reduction in cell cytotoxicity (Islam et al., 2021).

### *Streptococcus salivaruis*/*oralis* and *Staphylococcus epidermis*

*S. salivarius* and *S. oralis* are commensal α-hemolytic streptococci that are found in the human nasopharynx of healthy individuals. These commensals produce diffusible bacteriocin molecules such as Colcin V and exhibit pH lowering traits that inhibit biofilm formation and activity (Bidossi et al., 2018). In intranasal administrative studies these commensals were found to be safe and well tolerated, and to reduce biofilm formation associated with upper respiratory tract pathobionts including *S. aureus*, *S. pneumoniae*, and *M. catarrhalis* by up to 60% (Bidossi et al., 2018; De Grandi et al., 2019).

Some strains of *S. epidermis* secrete high levels of extracellular serine protease (Esp) and these strains are negatively associated with *S. aureus* nasal colonization (Iwase et al., 2010). Esp degrades proteins involved in biofilm formation and colonization, effectively disrupting *S. aureus* biofilms and leaving *S. aureus* susceptible to host antimicrobial peptides such as β-defensin-2 (Sugimoto et al., 2013). Inoculation of Esp producing *S. epidermidis* successfully eradicated *S. aureus* from human volunteers who had previously been consistently colonized (Iwase et al., 2010) validating it as a potential treatment option for future consideration. *S. epidermidis* also has immunomodulatory properties. When inoculated in a murine infection model with influenza A virus infected cells, increased IFN-γ production and suppressed replication of the virus was observed (Kim et al., 2019). Future studies should investigate *S. epidermidis in vivo* in determining its effectiveness in reducing severity of *S. aureus* and influenza A proliferation.

### Haemophilus haemolyticus

*H. haemolyticus* is a commensal URT bacteria which is phenotypically similar to *H. influenzae* and has historically been misidentified as such (Murphy et al., 2007). *H. haemolyticus* is capable of reducing *H. influenzae* attachment to epithelial cells (Pickering et al., 2016) and was recently shown to produce hemophilin (Latham et al., 2017, 2020). Hemophilin inhibits *H. influenzae* growth by binding to heme molecules that the pathobiont requires for growth (Latham et al., 2020). These mechanisms for competition and direct inhibition demonstrate *H. haemolyticus* has potential as a therapeutic probiotic.

It is clear that commensal microbiota in the URT have the potential to be used therapeutically to prevent pathobiont dominance and reduce the severity associated with dysbiosis in URT viral infections.

## Commensal Bacteria as Indirect Options for Prevention and Management of Upper Respiratory Viral Infections

The prevalence of upper respiratory infections signifies a key issue for the health care system, with annual costs in the billions (Fendrick et al., 2003). Some orally delivered probiotics, including bacteria derived from the human microbiota, have shown promising results for prevention or treatment of disease in both the gut and at other body sites (Hungin et al., 2018; Guo et al., 2019; Di Pierro et al., 2021). There is also some evidence that oral probiotics can help prevent or reduce the severity of URT infections (Hao et al., 2015; Wang et al., 2016). URT commensal bacteria have shown promise *in vitro* and may have potential as locally applied probiotics for the prevention and management of URT viral infections.

### Evidence and Therapeutic Use of Commensals in Clinical Setting

Manipulation of the microbiota can provide clinical benefits, including pathogen clearance and regulation of host immunity. For example, fecal microbiota transplantation has become the most effective treatment of recurrent *C. difficile* infection with a 91% success rate without recurrence of infection (Baunwall et al., 2020). Oral probiotics such as *Lactobacillus* and *Bifidobacteria* spp. have been shown to be beneficial for antibiotic induced diarrhea (Guo et al., 2019) and vaginal thrush (albeit with low evidence) (Xie et al., 2017). The mechanisms behind these effects include the production of antimicrobial compounds (Vieco-Saiz et al., 2019), altering the local environment to promote commensal growth, e.g., via acid production to lower pH (Islam, 2016), and effects on the host to increase immune tolerance via downregulation of inflammatory mediators (Gasta et al., 2017).

The natural oral probiotic, breast milk, has been associated with the development of a beneficial URT microbiota in infants (Lyons et al., 2020). However, probiotics delivered orally would be less likely to influence URT microbial outcomes long term in an older person with an established microbiota where there is reduced capacity for adherence and colonization (Esposito and Principi, 2018). Localized URT probiotics such as intranasal commensal inoculation would more likely have a greater inoculation rate and less systemic effects. *Corynebacterium* spp. and *D. pigrum* have many of the same features of currently used probiotics (pathogen inhibition, promotion of immune tolerance), indicating their potential use as localized sinonasal probiotics. In URTIs that are chronic or recurrent due to pathobiont dominance, transplantation of commensal species may be an option to reset the balance of the microbiota which could reduce incidence and severity of viral URTIs.

### Studies on Localized Probiotics for the URT

There are a limited number of studies regarding probiotics and their effect in URT disease. Some studies have focused on oral administration using innate commensal microbiota of the gastrointestinal tract such as *Lactobacilli* spp. and *Bifidobacterium* spp. This approach is predicated on the idea that gastrointestinal microbiota can influence the URT via systemic effects such as immune modulation, however, the evidence from these studies for influence on the URT is mixed (Hao et al., 2015; Li et al., 2020).

Localized URT therapies can be easily applied as a spray or rinse and several studies have explored this route of administration. *S. salivarius* and *S. mitis* have been trialed as nasal sprays and their use was associated with reduction of episodes of URT infections (Bellussi et al., 2018; Shekhar et al., 2019). Specifically, intranasal immunization of mice with *S. mitis* showed higher levels of IgG and IgA antibodies that are reactive to both *S. mitis* and *S. pneumoniae* resulting in reduced bacterial load of *S. pneumoniae* (Shekhar et al., 2019). The duration of these effects also needs to be considered. In a previous intranasal probiotic study a nasal spray containing 10^7^ CFU per spray of *S. sanguis*, *S. mitis*, and *S. oralis* (in equal amounts) was detected for up to 12 h in the nasopharynx but not after 36 h (Tano et al., 2002). Even with daily application there was no significant effect in reducing the number of episodes in sufferers of recurrent acute otitis media, and it was proposed by the authors that without antibiotics to remove the natural microbiota and create an available niche for the probiotic, that they would be unlikely to adhere and change the microbiota permanently.

Given the negative association of *Corynebacterium* spp. and *S. aureus* colonization, several *Corynebacterium* spp. have also been explored as a probiotic to remove *S. aureus* from the nose. A *Corynebacterium* sp. (Co304) was repeatedly inoculated into 17 healthy adults known to be colonized with *S. aureus* and found to eradicate *S. aureus* colonization in 12 of the participants, where controls of saline or *S. epidermidis* did not (Uehara et al., 2000). Similar results were seen in a smaller, uncontrolled study where inoculation of *C. pseudodiphtheriticum* was associated with removal of *S. aureus* from three out of four volunteers, and a reduction of *S. aureus* load in the fourth (Kiryukhina et al., 2013). This is likely to lead to the investigation of known commensals *in vivo* such as *Corynebacterium* spp. and *D. pigrum* that have both demonstrated significant favorable effects *in vitro*.

## Conclusion

It is possible that upper respiratory viral pathogens benefit from increased abundances of one or more pathobiont bacterial species, as is often observed in URT microbiota dysbiosis (Bosch et al., 2013). Given the ability of commensal URT bacterial species to inhibit the growth or colonization of pathobionts, manipulation of the microbiota could be utilized as a preventative or treatment strategy in combating upper respiratory viral infections. With external triggers, along with medication use including antibiotics and steroids likely influencing the URT microbiota, further research into innate and preventative therapies may benefit individuals with chronic respiratory diseases that rely on these medications. Pathobiont abundance is increased during symptomatic but not asymptomatic viral infection, suggesting that symptomatic viral infections may be prevented, or their severity reduced if commensal bacteria are applied to reduce or prevent pathobiont abundance (Chonmaitree et al., 2017).

The commensal bacteria described above that show the potential to inhibit pathobionts and modulate host immunity should be further studied for their potential to stimulate a resilient sinonasal microbiota that is resistant to URT viral infection. The development of *in vivo* and *in vitro* models that assess microbial competition and interactions within the microbiota will further our understanding of the complex relationships that exist and bring us closer to developing probiotic solutions for URT infections.

## Author Contributions

HN, CB, and MH wrote this literature review. All authors contributed to the article and approved the submitted version.

## Conflict of Interest

The authors declare that the research was conducted in the absence of any commercial or financial relationships that could be construed as a potential conflict of interest.

## Publisher’s Note

All claims expressed in this article are solely those of the authors and do not necessarily represent those of their affiliated organizations, or those of the publisher, the editors and the reviewers. Any product that may be evaluated in this article, or claim that may be made by its manufacturer, is not guaranteed or endorsed by the publisher.
